# Suppression of Metastatic Melanoma Growth in Lung by Modulated Electro-Hyperthermia Monitored by a Minimally Invasive Heat Stress Testing Approach in Mice

**DOI:** 10.3390/cancers12123872

**Published:** 2020-12-21

**Authors:** Mbuotidem Jeremiah Thomas, Enikő Major, Anett Benedek, Ildikó Horváth, Domokos Máthé, Ralf Bergmann, Attila Marcell Szász, Tibor Krenács, Zoltán Benyó

**Affiliations:** 1 Institute of Translational Medicine, Semmelweis University, 1094 Budapest, Hungary; major.eniko@med.semmelweis-univ.hu (E.M.); benedek.anett@med.semmelweis-univ.hu (A.B.); benyo.zoltan@med.semmelweis-univ.hu (Z.B.); 2 Department of Biophysics and Radiation Biology, Semmelweis University, 1094 Budapest, Hungary; horvath.ildiko@med.semmelweis-univ.hu (I.H.); mathe.domokos@med.semmelweis-univ.hu (D.M.); r.bergmann@hzdr.de (R.B.); 3 Hungarian Centre of Excellence for Molecular Medicine (HCEMM), In Vivo Imaging Advanced Core Facility, 1094 Budapest, Hungary; 4 Helmholtz-Zentrum Dresden Rossendorf, Institute of Radiopharmaceutical Cancer Research, 01328 Dresden, Germany; 5 Department of Internal Medicine and Oncology, Semmelweis University, 1083 Budapest, Hungary; szasz.attila_marcell@med.semmelweis-univ.hu; 6 1st Department of Pathology and Experimental Cancer Research, Semmelweis University, 1085 Budapest, Hungary; krenacs.tibor@med.semmelweis-univ.hu

**Keywords:** modulated electro-hyperthermia, B16F10 melanoma, pulmonary metastases, DNA double-strand breaks, cell cycle arrest, immune cell mobilization

## Abstract

**Simple Summary:**

The lung is the most frequent site of distant melanoma metastases. Metastases of melanoma in the lungs offer a very poor prognosis, with a 5-year survival rate of below 10%. Hyperthermic therapies including modulated electro-hyperthermia (mEHT) in clinical settings have been used to improve the efficacy of radiotherapy, chemotherapy, and immunotherapy of tumors. In this study, we focused primarily on the optimization of mEHT for targeted lung treatment of mice lungs burdened with B16F10 melanoma pulmonary metastases, with a particular focus on elucidating the mechanism of action of mEHT on treated melanoma cells while investigating any potential treatment-related side effects on normal lung tissue. mEHT showed evidence of significant anti-tumor effects as demonstrated by the reduced number of pulmonary metastatic nodules, DNA damage response, downregulation of Ki67 expression, higher immune cell infiltration, and upregulation of p21^waf1^ expression in mEHT-treated tumors.

**Abstract:**

Modulated electro-hyperthermia (mEHT) is a novel complementary therapy in oncology which is based on the higher conductivity and permittivity of cancerous tissues due to their enhanced glycolytic activity and ionic content compared to healthy normal tissues. We aimed to evaluate the potential of mEHT, inducing local hyperthermia, in the treatment of pulmonary metastatic melanoma. Our primary objective was the optimization of mEHT for targeted lung treatment as well as to identify the mechanism of its potential anti-tumor effect in the B16F10 mouse melanoma pulmonary metastases model while investigating the potential treatment-related side effects of mEHT on normal lung tissue. Repeated treatment of tumor-bearing lungs with mEHT induced significant anti-tumor effects as demonstrated by the lower number of tumor nodules and the downregulation of Ki67 expression in treated tumor cells. mEHT treatment provoked significant DNA double-strand breaks indicated by the increased expression of phosphorylated H2AX protein in treated tumors, although treatment-induced elevation of cleaved/activated caspase-3 expression was insignificant, suggesting the minimal role of apoptosis in this process. The mEHT-related significant increase in p21^waf1^ positive tumor cells suggested that p21^waf1^-mediated cell cycle arrest plays an important role in the anti-tumor effect of mEHT on melanoma metastases. Significantly increased CD3+, CD8+ T-lymphocytes, and F4/80+CD11b+ macrophage density in the whole lung and tumor of treated animals emphasizes the mobilizing capability of mEHT on immune cells. In conclusion, mEHT can reduce the growth potential of melanoma, thus offering itself as a complementary therapeutic option to chemo- and/or radiotherapy.

## 1. Introduction

Metastatic dissemination of cutaneous melanoma to subcutaneous tissues, locoregional lymph nodes, and distant organs and tissues is common. The lung is by far the most common site of distant metastases of melanoma. Lung metastases of melanoma predict a very poor prognosis, with a 5-year survival rate of less than 10% [1,2]. Several treatments have been approved by the United States Food and Drug Administration (FDA) in recent years for melanoma treatment. The treatment options may be surgical excision, radiotherapy, chemotherapy, photodynamic therapy, or immunotherapy, depending on the tumor’s characteristics, such as genetic profile, stage, and location [3]. Hyperthermic therapies, including modulated electro-hyperthermia (mEHT), have been used to enhance the efficacy of chemotherapy, radiation therapy, and, recently, immunotherapy of tumors [4,5,6,7]. mEHT capacitive coupling creates an electromagnetic field that is predominantly absorbed in the tumor relative to adjacent normal tissues [8]. Tumor cells demonstrate increased glucose uptake due to the Warburg effect, thus resulting in an enhanced buildup of lactate, accompanied by an increased metal ion and salt content [8,9,10,11], leading to increased conductivity and dielectric permittivity [12,13,14,15]. This increases the selective absorption of the electromagnetic field by tumors, leading to increased heat generation within the tumor mass. mEHT therapy has been demonstrated to induce substantial apoptosis and decreased tumor proliferation in glioma cultures [16]. In colorectal adenocarcinoma models, mEHT treatment has been demonstrated to induce both caspase-dependent and caspase-independent AIF-mediated apoptosis [17,18,19]. In triple-negative breast cancer isografts, exhaustion of mEHT-induced heat shock response resulted in significant tumor damage via a caspase-dependent mechanism in mice [20].

The effects of mEHT on lung cancers have not been studied before in a mouse model. This is probably due in part to the lack of appropriate devices optimized for targeted lung treatment without treatment-associated tissue damage of neighboring structures in the thorax such as the heart. In addition, lung temperature must be measured at all time points during treatment to ensure that the targeted treatment temperature is reached but not exceeded, as this may pose a significant risk of lung damage and/or necrosis. The measurement of lung temperature during treatment poses significant challenges as placement of even the tiniest optical thermosensor in the lungs may induce severe injury, bleeding, and possible pneumothorax, especially during repeated mEHT treatments of tumors. Furthermore, possible treatment-related adverse effects such as lung injury, inflammation, and the development of pathological fibrotic lesions with mEHT-treatment of lungs have not been investigated before.

B16 melanoma is a C57BL/6-derived, highly aggressive variant of melanoma with high metastatic capability from subcutaneous location to lungs. Intravenous injection of B16F10 melanoma cells readily results in metastatic colonization of the lungs [21]. This makes it a suitable tumor model for our experimental studies. In this study, we first focused on the optimization of mEHT for the targeted lung treatment of tumors. The development of an efficient electrode capable of inducing a localized increase in lung temperature with minimal heat dissipation to neighboring tissues and organs was our first priority. Subsequently, we developed a minimally invasive method of monitoring lung temperature during mEHT treatment without direct placement of optical thermosensors in mice lungs.

Furthermore, we investigated the effect of mEHT treatment on B16F10 melanoma pulmonary metastases growth with a particular focus on the cellular mechanisms of tumor growth inhibition, including DNA damage response, apoptosis, immune response, cell cycle arrest, and treatment-associated pathological changes in the lungs. We have demonstrated a significant anti-tumor effect in the lungs after six-times treatment with mEHT in C57BL/6 mice. Tumor growth inhibition was associated with a significant reduction in Ki67 tumor proliferation marker expression in treated animals. Increased DNA damage response was also observed in treated tumors, although this was not accompanied by a significant increase in cleaved caspase-3, suggesting that apoptosis plays only a minimal role in the anti-tumor effect of mEHT in vivo under the current treatment dose of this study. However, prolonged in vitro treatment of B16F10 melanoma cells showed significantly increased apoptosis and necrosis, indicating that cellular death by mEHT is largely dose-dependent and longer in vivo treatment other than the treatment duration discussed in this paper may offer higher therapeutic benefit. Furthermore, mEHT induced significant cell cycle arrest, as indicated by the increased expression of p21^waf1^, a cyclin-dependent kinase (CDK) inhibitor, in treated tumors. Increased immune cell density was observed in both the whole lungs and tumors of treated animals, suggesting that mEHT was capable of mobilizing the immune system, leading to significant infiltration of immune cells into treated lungs and tumors. The treatment-related adverse effects of mEHT on the lungs were also investigated. Although a significant increase in inflammatory infiltrates was observed under acute conditions, measured two days after the last mEHT treatment, insignificant levels of inflammatory infiltrate were measured under chronic conditions post-mEHT treatment. Likewise, no fibrotic lesions were observed in the lungs of treated animals in both acute and chronic conditions. The cumulative lung injury score was also evaluated, taking into consideration scores of several composing parameters, including cellular infiltrate, alveolar over-distension, atelectasis, interstitial congestion, and hemorrhage following mEHT treatment of non-tumor bearing lungs, to better characterize the full spectrum of potential mEHT-related possible adverse effects on normal lung tissue. Significant changes in lung injury were not observed in treated animals, indicating the absence of auxiliary damage to normal lung tissue that may be associated directly with mEHT.

## 2. Results

### 2.1. mEHT Induced Localized Increase in Lung Temperature

**Figure 1:**
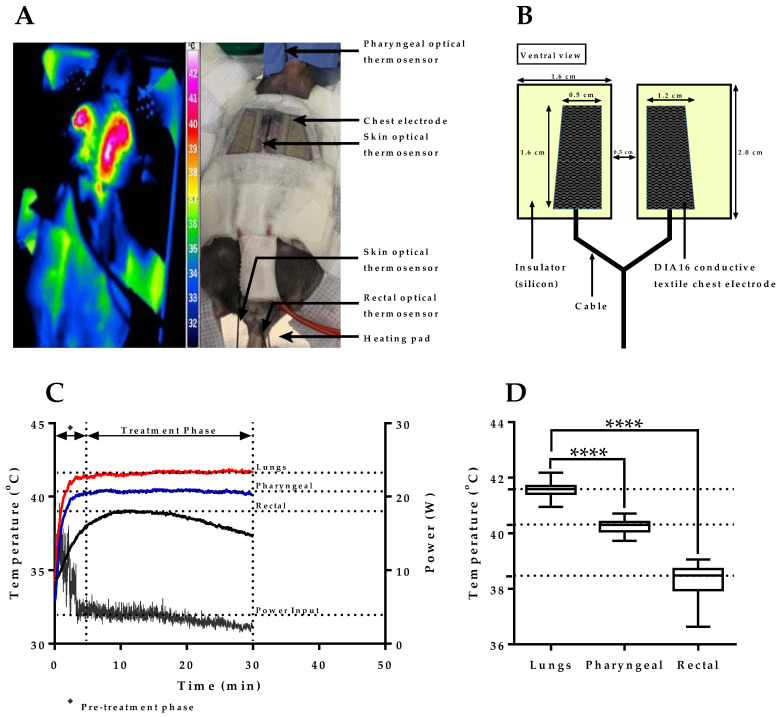
Localization of heat stress during targeted mEHT treatment of lungs. (**A**) Thermal image depicting localized increase in temperature over target area on mice thorax (**left** panel) and treatment layout with the positioning of chest electrode and optical thermosensors (**right** panel); (**B**) Dimensions of DIA16 conductive textile chest electrode for targeted lung treatment of tumors in mice; (**C**) Plot depicting temperature profiles of lungs, pharynx, and rectum with applied power settings during pilot experiment; (**D**) Pharyngeal and rectal temperature versus lung temperature in treatment phase (*n* = 4). Data represent average ± SEM. Mann–Whitney test, **** *p* < 0.0001.

As mEHT has not been used before for interventions in the lung, our first objective was to develop a suitable chest electrode capable of inducing lung-specific hyperthermia without treatment-associated damage to nearby structures such as the heart, liver, and skin in mice. Because the impedance of the lung to transmitted energy is far higher than that of the neighboring tissues, the electrode needed to cover exclusively the target area, thereby avoiding the dissipation of energy to nearby structures. Based on these considerations, several chest electrodes were designed and tested for inducing lung-specific hyperthermia with a targeted treatment temperature range of 41.5–42.0 °C, and the upper DIA16 conductive textile chest electrode shown in Figure 1A (right panel) was found to be optimal for lung treatment. Pilot experiments demonstrated a localized increase in temperature over the target area on mice chests as depicted by the externally measured thermal mapping shown in Figure 1A (left panel). Figure 1B depicts the dimensions of the DIA16 conductive textile chest electrode used in this study for the targeted treatment of lung tumors in mice. Figure 1C shows the power adjustment profile of mEHT in a pilot experiment with measured lung, pharyngeal, and rectal temperatures at different time points. A significant difference was observed between measured lung/pharyngeal and lung/rectal temperatures (*p* < 0.0001) with mean temperature values of 41.6 ± 0.1, 40.3 ± 0.1, and 38.5 ± 0.5 °C measured in the lung, pharynx, and rectum, respectively, during treatment (Figure 1D). These results demonstrate that the targeted temperature range for mEHT treatment of lungs could be achieved with the current chest electrode while maintaining limited dissipation of energy to nearby structures and the whole body.

### 2.2. Verification of Pharyngeal Temperature Measurement as a Minimally Invasive Method of Estimating Lung Temperature during mEHT Treatment of Lung Tumors

**Figure 2:**
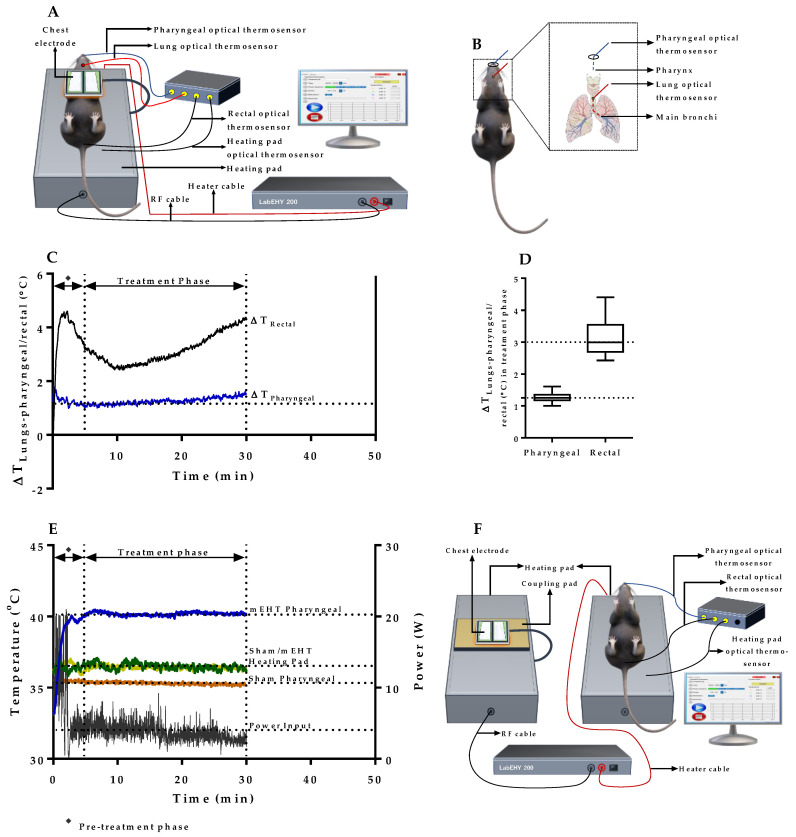
Experimental setup and quantitative relation of lung and pharyngeal temperatures during mEHT treatment of lungs. (**A**) Schematic illustration of the experimental setup of mEHT for lung treatment of mice. The illustration represents setup in pilot experiments for the establishment of quantitative relation between the lung and pharyngeal temperatures with thermosensors positioned in lungs, pharynx, rectum, and heating pad/lower electrode. In subsequent mEHT treatment of tumor-bearing lungs, thermosensors were placed only in pharynx, rectum, and heating pad/lower electrode; (**B**) Schematic illustration depicting placement of pharyngeal and lung optical thermosensors for the establishment of quantitative relation between the lung and pharyngeal temperatures in pilot experiments; (**C**) Temperature gradient between the lung and pharyngeal/rectal temperatures in pilot experiment (*n* = 4); (**D**) Plot of the mean difference between lungs and pharyngeal/rectal temperatures in pilot experiment (*n* = 4); (**E**) Plot depicting temperature profiles of the pharynx, and heating pad with applied power settings during mEHT treatment of tumor-bearing lungs; (**F**) Schematic illustration of sham treatment setup. Non-distilled, ion-containing water wet coupling pad was used as a coupling material for the emitted radiofrequency signal from the chest electrode. Data represent average ± SEM.

The direct measurement of lung temperature in mEHT is unsuitable as placement of optical thermosensor to the mouse lung may induce severe injury, bleeding, and possible pneumothorax, especially during repeated mEHT treatments of lung tumors. In a pilot experiment, optical thermosensors were advanced into the lung’s main bronchi, pharynx, and rectum of non-tumor-bearing animals and treated with mEHT for 30 min according to the experimental setup depicted in Figure 2A. Access to mice trachea was enabled via puncture tracheotomy, through which an optical thermosensor was advanced to the lung’s main bronchi and via oral access, and a pharyngeal optical thermosensor was placed in the pharynx of mice (Figure 2B). Lung temperature in the main bronchi was maintained at 41.5–42.0 °C using the power adjustment profile of mEHT and the pharyngeal temperature was monitored simultaneously. The objective here was to assess if a quantitative relation exists between the lung and pharyngeal temperature; in which case, if a relation is established, lung temperature could be estimated indirectly in subsequent experiments by measuring the pharyngeal temperature only. Figure 2C shows a plot of the temperature difference between lungs and pharyngeal/rectal with an observed minor deviation of the temperature difference seen in the pharynx. The minor deviation of temperature difference indicates the existence of a quantitative relation between the lung and the pharyngeal temperature based on which the lung temperature can be estimated from the pharyngeal temperature. The average temperature difference observed between the lungs and pharynx measured in the treatment phase (5–30 min) was 1.3 ± 0.1 °C (Figure 2D). This implies that by maintaining the pharyngeal temperature at 1.3 ± 0.1 °C lower than the targeted lung temperature of 41.5–42.0 °C, heat stress can be monitored and controlled less invasively in subsequent experimental treatment of lung tumors in mice. Following these pilot experiments, subsequent treatment of tumor-bearing lungs was conducted by measurement of the pharyngeal temperature only, as depicted in Figure 2E. In sham-treated, tumor-bearing animals, the chest electrode was placed on a coupling pad and pseudo-treated accordingly, while all other conditions were maintained the same as in mEHT-treated animals (Figure 2F). Taken together, these results demonstrate that pharyngeal temperature measurement during mEHT treatment is a viable method of estimating lung temperature and controlling heat stress generated in mice lungs during mEHT treatment of tumors without any need for direct lung temperature measurement with the associated risk of mechanical injury.

### 2.3. mEHT Induced Reduction of Metastatic Tumor Burden in Mice Lungs

**Figure 3:**
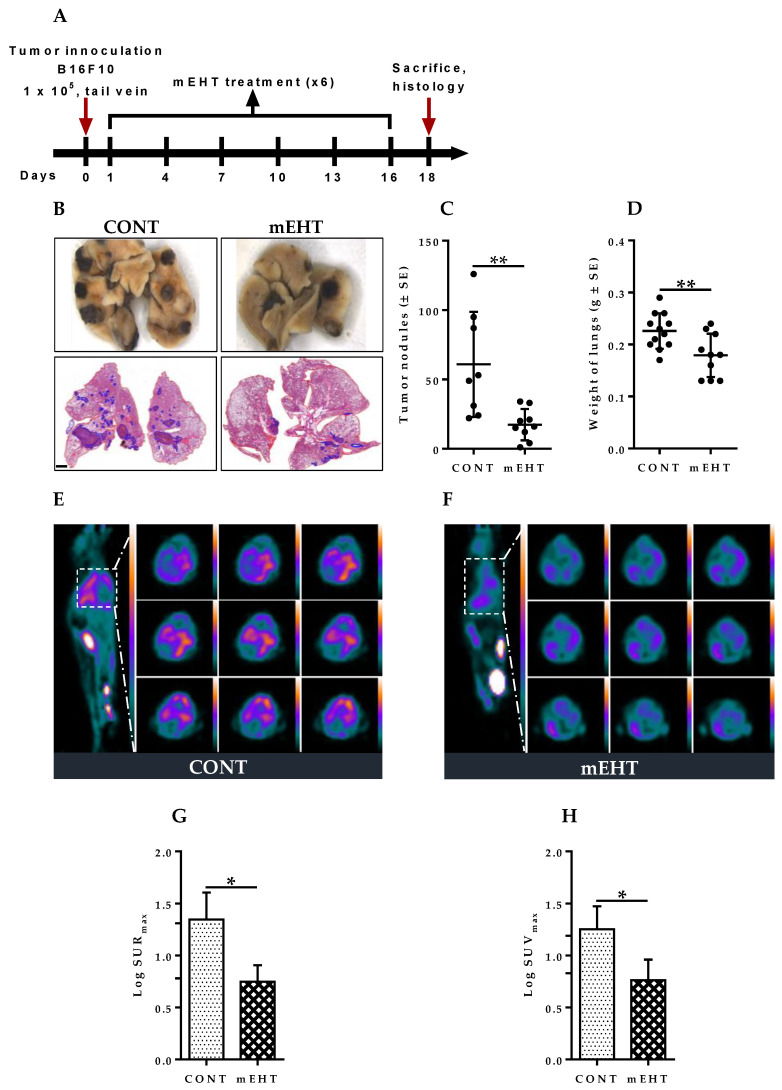
Effect of modulated electrohyperthermia on tumor burden in mouse lungs. (**A**) Schematic representation of the experimental protocol. Animals were injected with 1 × 10^5^ B16F10 melanoma cells via tail vein and treated six times with LabEHY-200; (**B**) Native and H&E stained lungs with pulmonary melanoma metastatic in CONT and mEHT-treated animals, (Scale bar, 2000 μm); (**C**) Quantification of pulmonary melanoma metastatic nodules in CONT (*n* = 8) and mEHT (*n* = 9); (**D**) Quantification of lung weight in CONT (*n* = 12) and mEHT (*n* = 10); (**E**,**F**) Representative PET images depicting [^18^F]FDG uptake in sagittal and coronal sections of CONT and mEHT; (**G**) Standardized uptake ratio (SURmax) of [^18^F]FDG in CONT (*n* = 4) and mEHT (*n* = 3); (**H**) Standardized uptake value (SUVmax) of [^18^F]FDG in CONT (*n* = 4) and mEHT (*n* = 3). Data represent average ± SEM. (**C**,**D**) CONT vs. mEHT, unpaired *t*-test, (**G**,**H**) CONT vs. mEHT, Mann–Whitney test. * *p* < 0.05, ** *p* < 0.01.

The lung is a common site of melanoma metastases [22]. To define a potential impact of mEHT in the host lung on the development of metastatic nodules, 1 × 10^5^ B16F10 melanoma cells were injected into the tail vein of seven-to-nine-week-old female C57BL/6 mice. In this study, we investigated the ability of mEHT to modulate the metastatic progression of melanoma as an aggressive tumor model that can easily metastasize to the lung. All animals were treated six times using the LabEHY-200 device (Oncotherm Ltd., Páty, Hungary). The first mEHT treatment of 30 min was performed one day after the tail vein injection of the animals with B16F10 melanoma cells. Treatment was repeated every third day for a total of six times as defined by the experimental protocol (Figure 3A). Visible tumor nodules were observed in the lungs of both mEHT-treated and sham-treated animals, indicating that metastatic tumors were successfully induced in mice lungs (Figure 3B). The pulmonary metastatic burden was assessed by counting the number of tumor nodules on the lung surface, 48 h after the last mEHT treatment on day 18. Mice in the mEHT-treated group displayed a significantly lower number of tumor nodules (average of 17), whereas numerous distinguishable melanoma nodules (average of 60) were observed in the lungs of sham-treated animals (*p* = 0.0049, Figure 3C). Furthermore, excised lungs were weighted to further characterize the difference in tumor burden between the treated and control group. As shown in Figure 3D, the mean lung weight in the mEHT-treated group was significantly reduced, indicating the suppression of tumor growth compared to the control group (*p* = 0.0090). Figure 3E,F show representative PET images depicting fluorodeoxyglucose ([^18^F]FDG) uptake in sagittal and coronal sections of mEHT-treated and sham-treated control. As depicted by Figure 3G,H, significantly reduced SURmax (*p* = 0.0169) and SUVmax (*p* = 0.0300) were observed in the mEHT-treated lungs compared to the sham-treated ones. Taken together, these results indicate that mEHT efficiently suppressed pulmonary melanoma growth and also reduced spontaneous lung metastasis.

### 2.4. mEHT Suppresses Melanoma Proliferation in the Lungs by Tumor Growth Inhibition without Direct Treatment-Induced Tumor Necrosis

**Figure 4:**
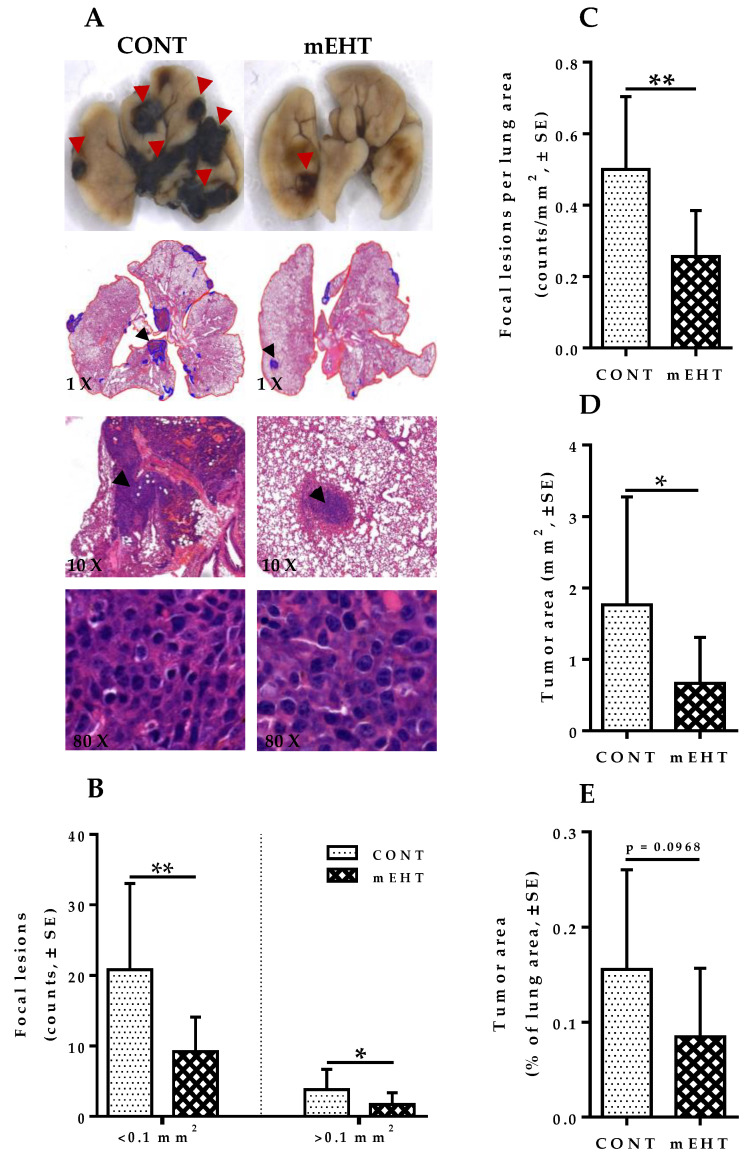
Inhibitory effect of modulated electrohyperthermia on tumor growth and proliferation. (**A**) Native and H&E-stained lungs with pulmonary melanoma metastases at higher magnification in CONT and mEHT-treated animals. (×80) magnification demonstrates the absence of any observable tumor necrosis after six-time treatment with mEHT; (**B**) Cumulative counts of focal metastatic melanoma lesions in CONT (*n* = 12) and mEHT (*n* = 10) sorted based on size (<0.1 and >0.1 mm^2^); (**C**) Cumulative counts of focal metastatic melanoma lesions per total lung area in CONT (*n* = 12) and mEHT (*n* = 10); (**D**) Tumor area occupied by melanoma in lungs in CONT (*n* = 12) and mEHT (*n* = 10); (**E**) Tumor area per total lung area in CONT (*n* = 12) and mEHT (*n* = 10). Data represent average ± SEM. unpaired *t*-test, * *p* < 0.05, ** *p* < 0.01.

Analysis of H&E-stained sections was performed to better characterize mEHT-related tumor growth suppression in the lungs, including intra-parenchymal tumors with no surface visibility, which often proves difficult to quantify by surface tumor nodules evaluation only. Digitalization of slides, annotation of tumor areas, evaluation of metastatic lesions counts, and viable tumor area per cross-sectional lung area was carried out using modules of the QuantCenter image analysis software tool pack (3DHISTECH). Multiple focal lesions with pleomorphisms and marked cellular atypia were observed in both mEHT-treated and sham-treated lungs. As Figure 4A (×80 magnification) indicates, no mEHT-induced direct tumor necrosis was observed in treated lungs. However, focal metastatic lesions in treated animals were significantly reduced in number, especially for lesions < 0.1 mm^2^ (*p* = 0.0032, Figure 4B). Likewise, the number of focal lesions >0.1 mm^2^ was significantly reduced in treated lungs compared to the sham-treated ones (*p* = 0.0497, Figure 4B). Additionally, counts per unit lung area of focal lesions showed a significant reduction in mEHT-treated animals compared to the sham-treated ones (*p* = 0.0039, Figure 4C). The total tumor area of focal metastatic melanoma lesions in mEHT-treated lungs was reduced significantly (*p* = 0.0383, Figure 4D), although the relative area occupied by these lesions per lung showed a marked reduction without reaching a statistically significant value (*p* = 0.0968, Figure 4E). Taken together, these results show that mEHT was capable of inducing significant tumor growth suppression, as demonstrated by the reduced number of metastatic lesions, tumor size, and the relative area occupied by lesions in the lungs. However, treatment-induced tumor necrosis was not observed in this study, which suggests that the tumor growth suppression by mEHT is largely inhibitory, especially of early spontaneous metastatic growth rather than direct heat-induced tumor necrosis.

### 2.5. mEHT Induced Reduction in Ki67 Expression in B16F10 Melanoma Cells

**Figure 5:**
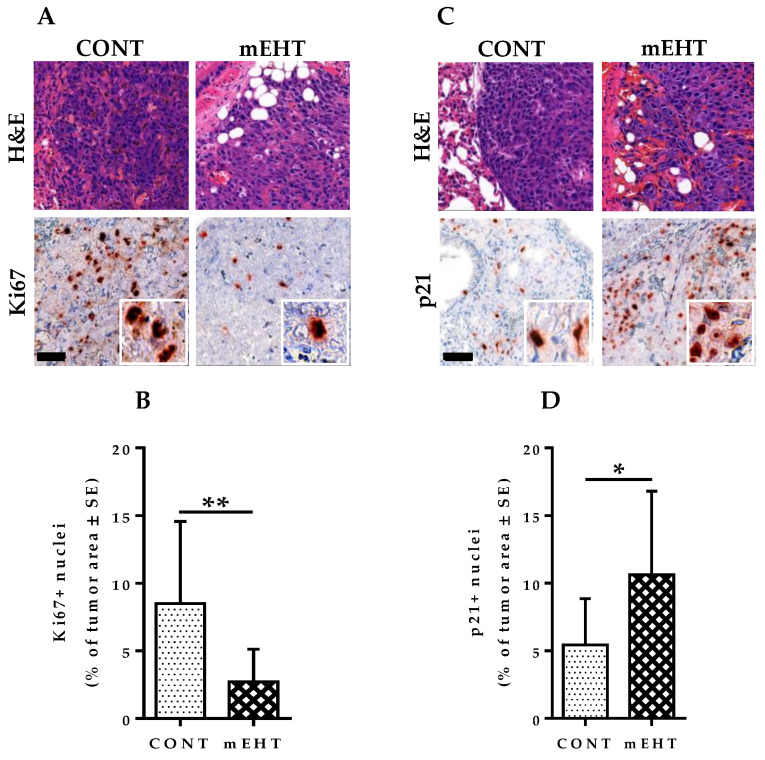
mEHT induced inhibition of tumor proliferation and cell cycle arrest. (**A**) H&E and Ki67 staining of tumor area in CONT and mEHT; (**B**) Plot depicting Ki67 positive nuclei as a percentage of total tumor area in CONT (*n* = 12) and mEHT (*n* = 10); (**C**) H&E and p21^waf1^ staining of mouse lungs in CONT and mEHT; (**D**) Plot depicting counts of p21^waf1^ positive nuclei as a percentage of total tumor area in CONT (*n* = 12) and mEHT (*n* = 10). Data represent average ± SEM. (**B**) CONT vs. mEHT, unpaired *t*-test, (**D**) CONT vs. mEHT, Mann–Whitney test, * *p* < 0.05, ** *p* < 0.01. Scale bar on (**A**,**C**) represents 50 μm on all low-power images and 15 μm in their insets.

The depletion of Ki67 in some human tumor cell lines and primary fibroblast cells has been demonstrated to slow entry into the S-phase and coordinately downregulate genes involved in DNA replication, thereby slowing the proliferation of tumor cells [23]. Therefore, we investigated whether Ki67 downregulation plays a role in mEHT-mediated inhibition of B16F10 melanoma growth. Ki67 expression was measured by immunohistochemistry with anti-Ki67 antibody in both mEHT-treated and sham-treated tumors (Figure 5A). Ki67 protein expression per unit tumor area was significantly reduced in mEHT-treated tumors compared to the control group (*p* = 0.0085, Figure 5B). This indicates the ability of mEHT to downregulate Ki67 protein expression in B16F10 melanoma cells and it explains the observed suppression of tumor growth seen in the treated lungs.

### 2.6. mEHT Induced Cell Cycle Arrest in B16F10 Melanoma Cells

Phosphorylated H2AX via the p53/p21^waf1^ pathway has been proven to play a major role in the induction of cell cycle arrest [24]. We have also demonstrated previously that mEHT treatment mediated p53 stabilization by acetylation of p53 in B16F10 melanoma cells [25]. p53 mediates the transcription of several proteins involved in cell cycle regulation, senescence, and apoptosis. One such protein is p21^waf1^, which is a cyclin-dependent kinase (CDK) inhibitor that promotes cell cycle arrest in response to a variety of stimuli. p21^waf1^’s inhibitory effect on the progression of the cell cycle coincides with its nuclear localization. Therefore, we investigated the effect of mEHT on the nuclear expression of p21^waf1^ in treated B16F10 melanoma cells. Samples from treated and sham-treated groups were stained for p21^waf1^ accordingly via immunohistochemistry and the quantity of positively stained cells per unit tumor area was quantified (Figure 5C). mEHT treatment led to significantly increased expression of p21^waf1^ positive melanoma cells in the treated group compared to the control (*p* = 0.0206, Figure 5D). Thus, we believe that p21^waf1^-mediated cyclin-dependent kinase inhibition leading to cell cycle arrest plays an important role in tumor growth inhibition by mEHT

### 2.7. mEHT Induced DNA Damage Response in Proliferating B16F10 Melanoma Cells without Significant Apoptosis

**Figure 6:**
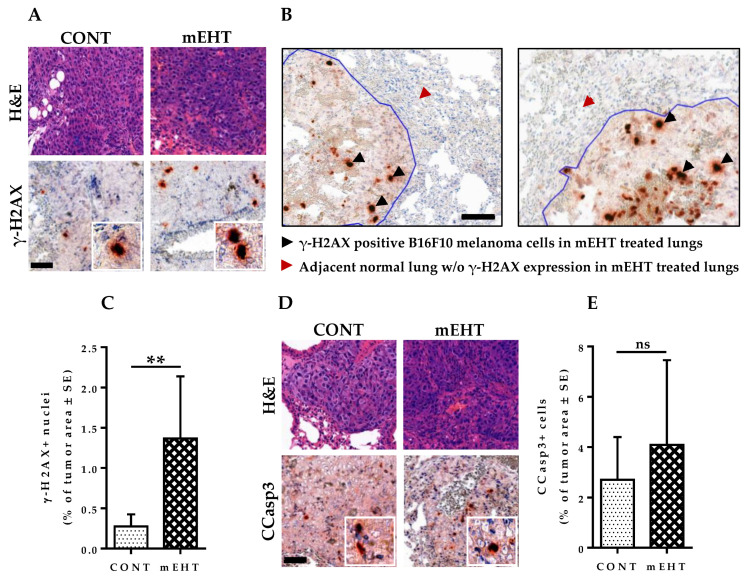
mEHT induced DNA damage response in metastatic B16F10 melanoma. (**A**) H&E and γ-H2AX staining of mouse lungs in CONT and mEHT; (**B**) Image depicting DNA damage response to mEHT treatment in tumor area but not in adjacent normal lung tissue; (**C**) Plot of γ-H2AX-positive nuclei as a percentage of total tumor area in CONT (*n* = 6) and mEHT (*n* = 7); (**D**) H&E and CCasp3 staining of mouse lungs in CONT and mEHT; (**E**) Plot of CCasp3-positive cells as a percentage of total tumor area in CONT (*n* = 12) and mEHT (*n* = 9). Data represent average ± SEM. (**C**,**E**) CONT vs. mEHT, unpaired *t*-test, ns = non-significant, ** *p* < 0.01. Scale bar on (**A**,**D**) represents 50 μm on all low-power images and 15 μm in their insets and 50 μm on (**B**).

Cellular response to DNA damage leads to a series of events, one of which is the phosphorylation of H2AX [26]. H2AX phosphorylation typically occurs immediately after a DNA break formation, resulting in the recruitment of clusters of DNA damage response proteins at the site of damage, forming a DNA damage response focus [27]. We tested the expression of phosphorylated H2AX (γ-H2AX) proteins to investigate whether mEHT induces significant DNA damage in treated tumors. DNA damage was detected by immunohistochemistry with an antibody against γ-H2AX and then analyzed using modules of the QuantCenter image analysis software tool pack (3DHISTECH) (Figure 6A). While treated tumors showed marked DNA damage response to mEHT-treatment, adjacent normal lung tissue did not demonstrate any visible signs of DNA damage, reiterating that mEHT-associated DNA damage is confined only to the highly proliferating melanoma cells, without associated damage to normal lung tissue (Figure 6B). In treated tumors, the expression of γ-H2AX was significantly increased after six-time treatment with mEHT compared to the sham-treated group (*p* = 0.0061, Figure 6C). Additionally, as DNA damage may lead to either cell senescence or programmed cell death [24], we investigated the role of apoptosis in mEHT-mediated inhibition of tumor growth and proliferation. Treated B16F10 melanoma cells demonstrated a comparable level of cleaved caspase-3 expression per tumor area to sham-treated ones, with no significant difference between both groups (*p* = 0.2331, Figure 6D,E). These results demonstrate the important role that DNA damage response induced by mEHT plays in limiting tumor growth and the proliferation of B16F10 melanoma cells while indicating the insignificant role that apoptosis plays in this process under the current treatment duration and conditions. Although a significant increase in DNA damage response has been observed in mEHT-treated tumors, the lack of mEHT-induced cell death at the current treatment dose suggests the possibility of cellular level pro-survival gene mutation of treated tumor cells.

### 2.8. mEHT Induced Cellular Death Is Dose-Dependent in B16F10 Melanoma Cells

**Figure 7:**
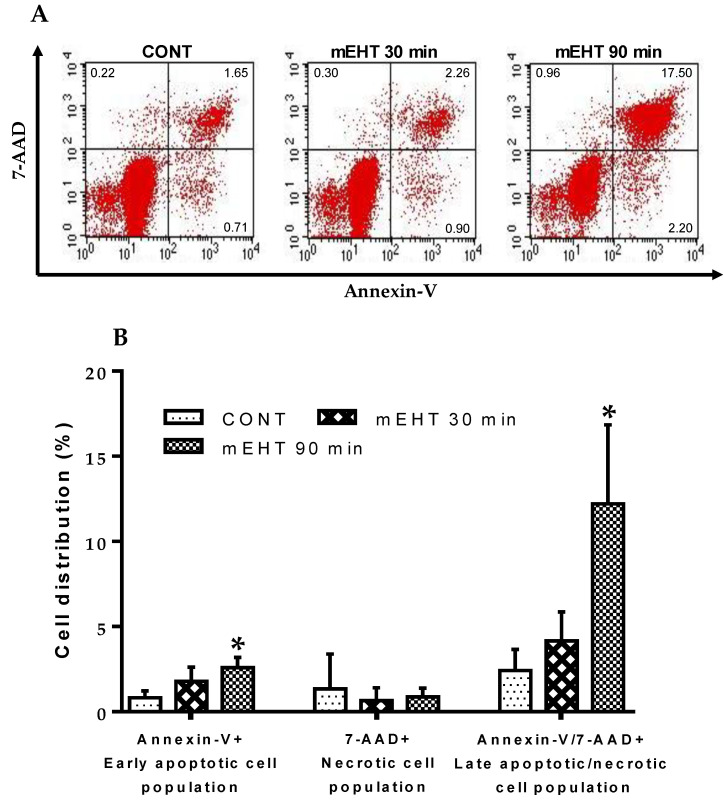
Dose-dependent induction of apoptosis and necrosis by mEHT in B16F10 melanoma cells. Tumor cells were mEHT-treated in vitro for the indicated duration of 30, 90 min. (**A**) Representative flow cytometry profiles for CONT and mEHT-treated B16F10 melanoma cells at 30 and 90 min treatment duration; (**B**) Dose effect of mEHT on cell death. Data represent average ± SEM of three independent experiments. CONT vs. mEHT (30, 90 min), unpaired *t*-test, * *p* < 0.05.

To investigate whether the observed lack of apoptosis or necrotic cell death after 30 min in vivo treatment in mice, as discussed in previous sections, was in any way influenced by the treatment dose, we conducted in vitro treatment of B16F10 melanoma cells for different treatment durations. B16F10 melanoma cells were treated in vitro for 30, 90 min, and cells were stained with Annexin-V and 7-AAD and analyzed using fluorescence-activated cell sorting (FACS). Figure 7A depicts representative flow cytometry profiles for control and mEHT-treated B16F10 melanoma cells at 30, 90 min treatment duration. In Figure 7B, percentage cell distribution as measured by FACS is represented for control and mEHT-treated B16F10 melanoma cells. Although 30 min treatment with mEHT did not yield any significant changes (*p* = 0.1410) in the early apoptotic cell population (Annexin-V+/7-AAD−), this was significantly increased after 90 min treatment compared to the control group (*p* = 0.0134). By contrast, significant changes in strictly necrotic (Annexin-V-/7-AAD+) cell population were not observed after both 30 and 90 min treatments in both mEHT-treated and control groups. Double positive late apoptotic/necrotic (annexin-V+/7-AAD+) cells were demonstrated to be significantly elevated after 90 min treatment (*p* = 0.0241) but not after 30 min treatment (*p* = 0.2287). Taken together, these results indicate that apoptosis and necrotic cell death may play an important role as the underlying cell death type, especially in prolonged mEHT treatment of B16F10 melanoma cells. However, this effect is dose-dependent and whether prolonged in vivo mEHT treatment beyond 30 min, as currently described in this paper, holds a similar benefit is yet to be investigated.

### 2.9. T-Lymphocytes and Antigen-Presenting Cells Are Increased in mEHT-Treated Lungs and Tumors

**Figure 8:**
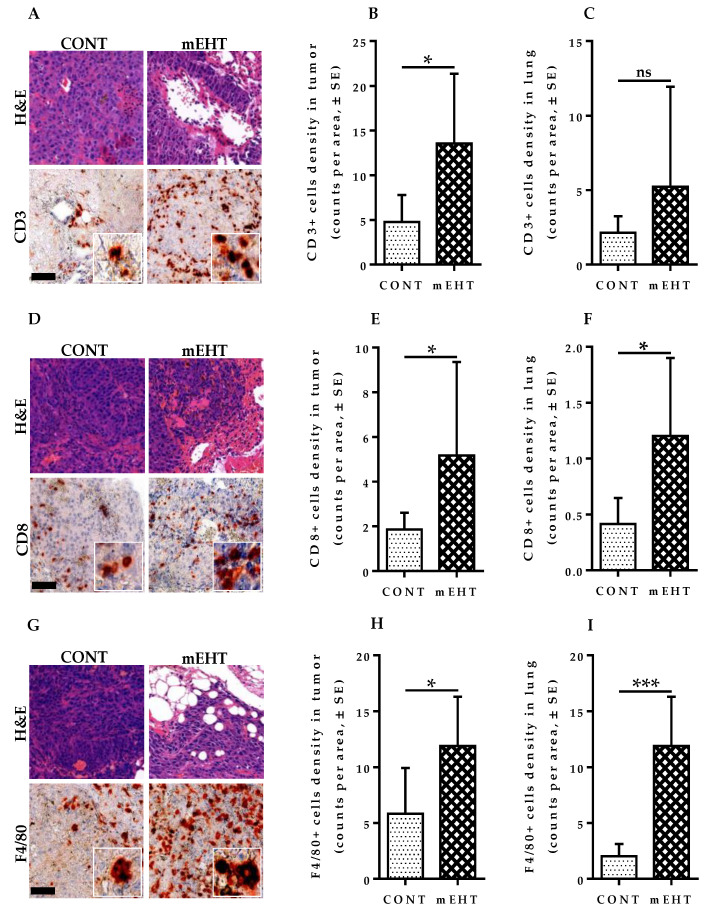
mEHT induced immune cell infiltration in focal metastatic melanoma lesions. (**A**) H&E and CD3 staining of mouse lungs in CONT and mEHT; (**B**) Plot depicting CD3-positive cell density in tumor area in CONT (*n* = 6) and mEHT (*n* = 7); (**C**) Plot depicting CD3-positive cell density in total lung area in CONT (*n* = 6) and mEHT (*n* = 7); (**D**) H&E and CD8 staining of mouse lungs in CONT and mEHT; (**E**) Plot depicting CD8-positive cell density in tumor area in CONT (*n* = 6) and mEHT (*n* = 7); (**F**) Plot depicting CD8-positive cell density in total lung area in CONT (*n* = 6) and mEHT (*n* = 7); (**G**) H&E and F4/80 positive cell staining of mouse lungs in CONT and mEHT; (**H**) Plot depicting F4/80 positive cell density in tumor area in CONT (*n* = 5) and mEHT (*n* = 6); (**I**) Plot depicting F4/80 positive cell density in total lung area in CONT (*n* = 5) and mEHT (*n* = 6). Data represent average ± SEM. (**B**,**F**,**H**,**I**) CONT vs. mEHT, unpaired *t*-test, (**C**,**E**) CONT vs. mEHT, Mann–Whitney test, ns = non-significant, * *p* < 0.05, *** *p* < 0.001. Scale bar represents 50 μm on all low-power images and 15 μm in their insets.

To study the putative contribution of immune cells to tumor growth inhibition by mEHT, we hypothesized that mEHT treatment might result in an alteration in the immune status of the lungs and tumor microenvironment. To test this assumption, we analyzed the expansion of CD3+ T-cells, CD8+ T-cells, and F4/80+CD11b+ macrophage in the lungs of tumor-bearing mice using immunohistochemistry in mEHT-treated and sham-treated animals. Whole lung and tumor infiltrations were analyzed using modules of the QuantCenter image analysis software tool pack (3DHISTECH). Significantly higher CD3+ T-cell infiltration into mEHT-treated tumors was observed compared to sham-treated ones (*p* = 0.0252, Figure 8A,B). However, total lung CD3+ T-cell density was comparable between both groups, with no significant difference (*p* = 0.6037, Figure 8C). The differences were significant for CD8+ T-cells, a prominent subtype of CD3+ cells with higher expression of positive cells seen in tumors of mEHT-treated lungs (*p* = 0.0221, Figure 8D,E). The total lung CD8+ T-cell density was significantly increased as well in mEHT-treated mice (*p* = 0.0238, Figure 8F) compared to the control group. Furthermore, we investigated the density of F4/80+CD11b+ macrophages in the tumor and whole lung of mEHT-treated and sham-treated animals. A significantly higher density of F4/80+CD11b+ macrophages was observed in the tumors (*p* = 0.0363, Figure 8G,H) and whole lung (*p* = 0.0007, Figure 8I) of mice treated with mEHT compared to sham-treated animals. Taken together, these results demonstrate the ability of mEHT to initiate a higher immune response against B16F10 melanoma cells. However, whether the infiltrating immune cells are in their suppressed or fully activated state, which may lead to tumor destruction, is yet to be investigated.

### 2.10. mEHT-Treated Lungs Showed No Auxiliary Lung Damage after Six-Time Treatment

**Figure 9:**
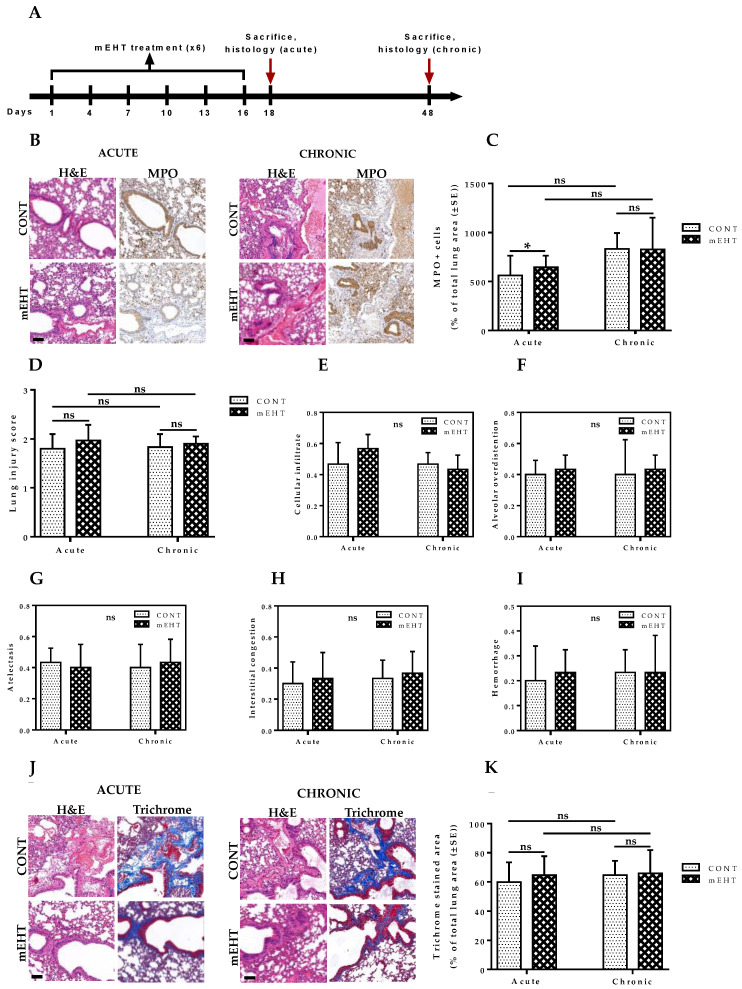
Acute and chronic treatment effect of mEHT on lung inflammation, lung injury, and pulmonary fibrosis. (**A**) Schematic representation of the experimental treatment protocol of non-tumor-bearing mice. Animals were treated six times with LabEHY-200 and sacrifice was performed 2 days after last mEHT treatment for acute and 32 days after last treatment for chronic evaluation of treatment-induced lung inflammation, lung injury, and fibrosis; (**B**) H&E and myeloperoxidase (MPO) staining of mouse lungs 2 days (acute) and 32 days (chronic) after last mEHT treatment in CONT and mEHT; (**C**) Plot of MPO-positive cells as a percentage of total lung area in CONT (*n* = 5) and mEHT (*n* = 5); (**D**) Cumulative lung injury score in CONT (*n* = 5) and mEHT (*n* = 5); (**E**–**I**) Component parameters of lung injury score which is a combined score of cellular infiltrate (0–4), alveolar over-distension (0–4), atelectasis (0–4), interstitial congestion (0–4), and hemorrhage (0 or 1). (**J**) H&E and Masson trichrome staining of mouse lungs 2 days (acute) and 32 days (chronic) after last mEHT treatment in CONT and mEHT; (**K**) Plot of Masson’s trichrome positive area as a percentage of total lung area in CONT (*n* = 5) and mEHT (*n* = 5). Data represent average ± SEM. (**C**,**D**,**K**) CONT vs. mEHT, unpaired *t*-test, (**E**–**I**) CONT vs. mEHT, Mann–Whitney test, ns = non-significant, * *p* < 0.05. Scale bar in B and J represents 100 μm.

Compared to conventional hyperthermia, mEHT can induce a significantly higher level of reactive oxygen species (ROS) in treated tumor cells [28]. While this offers obvious therapeutic benefits, such as ROS-mediated DNA damage of tumor cells [29], in normal lung tissues, ROS can activate NF-κB signal transduction, leading to the expression of a variety of inflammatory genes for cytokines, IL-4, IL-5, IL-9, IL-15, and TNF-α, with the potential for significant lung parenchymal damage [30]. Therefore, we investigated whether mEHT treatment of lungs can induce significant treatment-related lung parenchymal injury under acute and chronic conditions. Heat stress was induced by exposing the anesthetized non-tumor-bearing mice to a lung temperature of 41.5–42.0 °C for 30 min with mEHT. All mice were treated a total of six times, as described in the experimental protocol (Figure 9A). Non-tumor-bearing mice were selected for these studies to eliminate all potential influences that may affect the inflammatory status of the lungs. In two groups (sham-treated, mEHT-treated) of ten animals each, half of the animals in each group were sacrificed 48 h after the last mEHT treatment on day 18, and lungs were excised en bloc for further immunohistochemistry analysis. The remaining animals were kept under normal conditions for an extended 30-day period, after which sacrifice was made and lungs excised for further analysis.

All samples were myeloperoxidase-stained. Myeloperoxidase (MPO) is a heme-containing protein found mostly in azurophilic granules of neutrophils and, to a lesser extent, in monocytes [31]. Neutrophils undergo secondary necrosis during extensive inflammation, causing the release of MPO that may destroy resident lung cells [31]. Therefore, to characterize the potential inflammatory effect of mEHT on treated lungs, MPO positivity was measured via immunohistochemistry in both mEHT-treated and sham-treated animals and all samples were analyzed using modules of the QuantCenter image analysis software tool pack (3DHISTECH) to quantify the number of MPO-expressing cells (Figure 9B). As expected, mEHT induced a significant increase in MPO-positive cells under acute conditions compared to the control group (*p* = 0.0485). However, chronic levels of MPO-expressing cells measured on day 48, 32 days after the last treatment, showed no significant difference between both groups (*p* = 0.2730), suggesting that the inflammatory impact of mEHT on the lungs is restricted to acute conditions only (Figure 9C).

Furthermore, to understand the full spectrum of potential mEHT-induced adverse effects on treated lungs, we evaluated the lung injury score in both treated and sham-treated lungs, taking into consideration several composing parameters including cellular infiltrate, alveolar over-distension, atelectasis, interstitial congestion, and hemorrhage. The lung injury score was taken as a sum of the scores of the composing parameters. Significant changes between treated and sham-treated lungs were not observed, indicating the absence of auxiliary lung damage that may be associated with mEHT treatment (Figure 9D–I).

As inflammation can lead to fibrosis, especially under chronic conditions, we assessed the possibility of mEHT-induced fibrotic changes in the lungs by measuring the collagen contents in treated and sham-treated lungs. All samples were Masson’s trichrome-stained to characterize the collagen contents within the lungs (Figure 9J). As Figure 9K indicates, we did not find any evidence of increased collagen content in the lungs of mEHT-treated animals compared to sham-treated ones in both acute (*p* = 0.5381) and chronic conditions (*p* = 0.8927). Taken together, these results demonstrate that although mEHT was capable of inducing an acute increase in inflammatory cell infiltrates, this did not lead to any observable lung parenchyma damage, as demonstrated by the insignificant changes in lung injury score between treated and sham-treated animals. The absence of chronic inflammation in the lungs of treated animals explains the observed absence of fibrotic pathology and emphasizes the safety and lack of auxiliary lung damage that could limit the use of mEHT for the treatment of lung metastases.

## 3. Discussion

The tumor-damaging effect of mEHT has been well demonstrated in primary solid tumor models and clinical studies, the majority of which is used as a complementary therapy in combination with other treatment modalities [32,33,34,35]. Our primary objective for this study was the optimization of mEHT for the targeted treatment of lung tumors, as well as to elucidate the mechanism of action of mEHT treatment on B16F10 melanoma pulmonary metastases in mice, while investigating the potential side effects of the treatment on normal lung tissue.

First, multiple chest electrodes with different specifications were designed and tested for the capability of inducing a targeted increase in lung temperature without treatment-associated damage to neighboring tissues and organs in mice. The conventional electrode for solid tumor treatment was inappropriate for lung treatment due to insufficient contact with the thoracic surface and large size, which led to significant damage of adjacent tissues such as heart and liver during treatment. Next, electric parameters were set up in the pilot experiment to ensure a temperature of 41.5–42.0 °C in the lungs during treatment by using invasive temperature monitoring with optical thermosensors positioned in the main bronchi of mice lungs, pharynx, and rectum of non-tumor-bearing animals for the simultaneous measurement of temperatures during mEHT treatment. These pilot experimental results revealed the existence of a standard temperature gradient between the lungs and pharynx of mice, with an average temperature difference of 1.3 ± 0.1 °C, based on which subsequent mEHT treatment of tumor-bearing mice was conducted by maintaining the pharyngeal temperature at 1.3 ± 0.1 °C lower than the targeted lung temperature of 41.5–42.0 °C, without direct placement of optical thermosensors in the lungs. This technique, we believe, could offer a particular advantage in clinical settings where accurate estimation of lung temperature is essential to eliminate over-treating or under-treating of primary or metastatic pulmonary cancers, which may have a potential negative impact on the outcome of treated patients.

Serial treatment of the lungs with the newly developed method demonstrated significant inhibition of tumor growth, leading to reduced tumor burden in mEHT-treated animals. This was demonstrated by an apparent reduction in pulmonary surface melanoma nodules and lung weight when compared to sham-treated animals. SUVmax and SURmax as measured by PET showed significantly reduced average values in mEHT-treated animals, emphasizing the tumor inhibitory effect of mEHT on melanoma growth in the lungs.

Furthermore, analysis of H&E-stained lungs was done to further characterize the effect of mEHT on B16F10 melanoma growth in the lungs, including intraparenchymal nodules with no surface extension. Although the direct tumor-damaging effect of mEHT on tumors was not observed, a significant reduction in the number of metastatic melanoma lesions per cross-sectional lung area in treated animals was apparent.

Metastatic lesion count was reduced significantly after six-time mEHT treatment compared to the control group. Tumor size was reduced significantly in mEHT-treated lungs compared to sham-treated ones. As no direct tumor necrotic effect of mEHT was observed in treated tumors, a plausible explanation for the obvious decrease in tumor burden seen in treated animals is that the mEHT effect on B16F10 metastatic melanoma is mainly growth inhibition rather than direct heat-mediated tumor necrosis. One possible explanation for the observed inhibitory effect of mEHT on melanoma is the downregulation of Ki67 protein expression in treated tumors, which was accompanied by a comparable decrease in tumor size in treated lungs. Therefore, we believe that this plays a significant role in the observed effect of mEHT on melanoma.

Many events that cause either cell cycle arrest or apoptosis are associated with increased phosphorylation of H2AX in the cellular response to DNA damage. We measured the level of γ-H2AX in treated and sham-treated lungs to investigate the DNA damaging effect of mEHT. Treated lungs showed a significantly elevated level of γ-H2AX expression in tumor cells compared to the sham-treated control. Increased H2AX phosphorylation is linked to p53 protein upregulation associated with increased p21^waf1^, a strong cyclin-dependent kinase inhibitor [24]. We and other groups have reported that mEHT treatment alone induced caspase-independent AIF-mediated and caspase-dependent apoptosis in a colorectal adenocarcinoma model [36,37,38]. Glioma cultures also showed significant apoptosis and reduced tumor proliferation by mEHT [16]. In the present study and under the current treatment duration and conditions, however, mEHT treatment did not lead to a significant induction of apoptosis, as demonstrated by the insignificant levels of CCasp3 expression in treated tumors in vivo. We hypothesized that mEHT-dependent cellular death via apoptosis and/or necrosis may be dose-dependent; therefore, we investigated whether longer treatment time, beyond the 30 min used in the present study, can lead to increased cellular death by mEHT in vitro. Indeed, we observed that prolonged mEHT treatment of B16F10 melanoma cells for 90 min in vitro resulted in significantly increased apoptosis and necrosis, while in vitro treatment for 30 min did not show any evidence of increased cellular death as measured by FACS. This confirms the observed lack of apoptosis and necrotic cell death with mEHT treatment of pulmonary metastases in mice under the current treatment dose of 30 min and indicates that mEHT-induced cellular death is dose-dependent. Thus, longer in vivo treatment of B16F10 melanoma other than the treatment dose discussed in this paper may offer a higher therapeutic advantage.

The control of cell cycle progression is mediated by several factors in response to multiple stimuli, amongst which p21^waf1^, a potent cyclin-dependent kinase (CDK) inhibitor has been widely described. The nuclear localization of p21^waf1^ in response to external or internal stimuli correlates with its inhibitory effect on cell cycle progression [33]. We, therefore, investigated if mEHT induces a significant level of p21^waf1^ nuclear localization in treated tumors. Indeed, p21^waf1^ expression and nuclear localization were upregulated in treated tumors, suggesting that p21-mediated cell cycle arrest may play an important role in the inhibitory mechanism of mEHT. Marked elevation and nuclear localization of p21^waf1^ leading to senescence and cell cycle arrest account for a significant portion of the inhibition of tumor growth, which results in senescence of tumor cells. These cells are typically eliminated by phagocytes, whose activity is increased with hyperthermia treatment [34]. We, therefore, measured the level of F4/80+CD11b+ macrophage infiltrates in treated tumors to investigate the contribution of macrophages to the clearance of dead or senescent tumor cells. As expected, mEHT-treated lungs showed significantly increased F4/80+CD11b+ macrophage density in both tumors and whole lungs, indicating the critical role that these cells may play in the mEHT treatment.

A major determinant of tumor response to treatment is the level of immune response activated by the dying cancer cells. Therefore, we investigated the lymphocytic profile in treated and sham-treated tumors to increase our understanding of the immune mobilizing nature of mEHT treatment. As anti-tumor immunity is, in most cases, predominantly mediated by cellular immunity, we measured the quantity of the whole lung and tumor-infiltrated number of CD3+- and CD8+-T cells. We observed markedly increased CD3+-T cells in treated tumors, and although an increase in CD3+-T cell density in the lung as a whole was detected, the difference was statistically insignificant between both groups. An apparent increase in the whole lung and tumor density of CD8+-T cells was observed in treated animals. Although mEHT treatment resulted in significantly increased T cell infiltration into treated tumors, it is yet to be investigated whether the infiltrating lymphocytes are suppressed or actively involved in the tumor destruction of B16F10 melanoma cells.

Furthermore, our investigation of the potential treatment-associated side effects of mEHT on normal lung tissue did not yield any significant changes in the number of inflammatory cell infiltrates or fibrotic lesions under chronic conditions in treated animals, although an acute increase in inflammatory cell infiltrates was observed. However, this did not lead to any significant lung injury, as demonstrated by the insignificant cumulative injury score that took into consideration the impact of mEHT on cellular infiltrate, alveolar over-distension, atelectasis, interstitial congestion, and hemorrhage in the lungs.

## 4. Materials and Methods

### 4.1. Cell Culture

B16F10 mouse melanoma cell line (ATCC^®^ CRL 6475™) was purchased from ATCC (Manassas, VA, USA). Cells were cultured in minimum essential medium (MEM) supplemented with 1% (*v*/*v*) MEM-vitamin solution, 5% (*v*/*v*) heat-inactivated HyClone fetal bovine serum, 1 mM sodium pyruvate, 2 mM L-glutamine, and 1% (*v*/*v*) nonessential amino acids (NEAAs), purchased from Thermo Fisher Scientific (Waltham, MA, USA).

### 4.2. PET Imaging and Data Analysis

All procedures were carried out according to the guidelines of the Hungarian Law of Animal Protection (28/1998) and were approved by the Government Office of Pest County (Permission number: PE/EA/50-2/2019 and PE/EA/51-2/2019). Animals tested in this study were the offspring of C57BL/6 colonies grown in the animal facility of the Semmelweis University. In vivo PET experiments were conducted at the Department of Biophysics and Radiation Biology, Semmelweis University. Animals were fasted before the experiment for 6 h. Prior to PET imaging, 12–18 MBq radioactivity of [^18^F]FDG (FDG-KEDOI^®^ injection, Pet-Medicopus Ltd., Hungary) in 0.2–0.3 mL was injected into the tail vein of the animals. After 90 min of post-injection awake [^18^F] FDG uptake period, animals were anesthetized with 1.5% isoflurane in medical oxygen. During anesthesia, animals were placed in an immobilizing animal bed (MultiCell, Mediso, Hungary). Static, 15 min PET whole-body scans of animals were performed using a microPET P4 scanner (CTI Concorde Microsystems, Knoxville, TN, USA). Image volumes from the collected list mode data were then reconstructed using the instrument’s dedicated three-dimensional maximum a posteriori algorithm with a 1.56 mm voxel size. Data evaluation was carried out using two dedicated small animal image analysis software programs, Fusion (Mediso, Hungary) and vivoQuant (inviCRO, Boston, MA, USA). Three-dimensional lung regions of interest were drawn using a connected threshold algorithm, while blood regions of interest were manually drawn around the left ventricle of the heart. For the calculation of the Tumor Maximal Standardized Uptake Ratio (SURmax), and SUVmax, the standardized uptake values (SUV) were estimated by the following equation: [tissue concentration (MBq/mL) × the body weight (g)]/injected dose (MBq). Thereafter, SURmax was obtained by time correcting the ratio of lung maximal SUV value in the lung region of interest (SUVmax) to blood SUV multiplied by the ratio of 75 min post-injection to 90 min post-injection, as described by Hofheinz et al. [35,36,37]. After calculations, all mouse PET quantitative data were normalized to the respective lung mass (in grams) of each animal to account for initial size-related vascularization-derived differences of metastatic burden.

### 4.3. In Vivo mEHT Treatment

**Figure 10:**
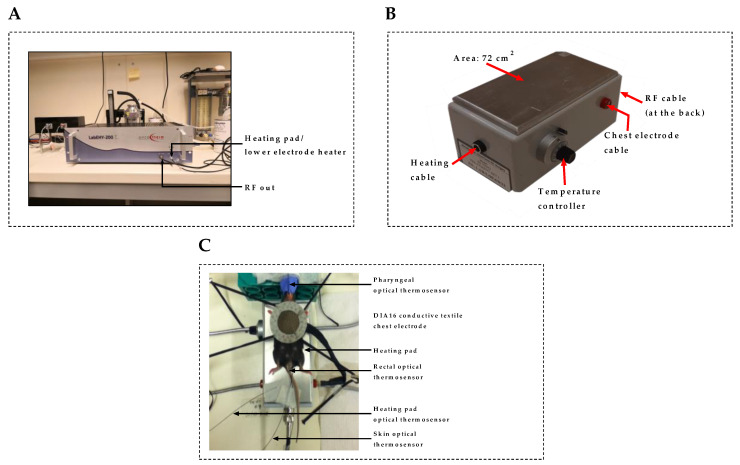
Treatment devices of modulated electrohyperthermia in mice. (**A**) LabEHY-200 treatment device; (**B**) Heating pad/lower electrode; (**C**) Conventional round upper electrode with main application in the treatment of primary solid tumors.

Pulmonary metastases of melanoma were established by injecting 10^5^ B16F10 melanoma cells via tail vein into seven-to-nine-week-old female C57BL/6 mice. The animals were treated six times using the LabEHY-200 device (Oncotherm Ltd., Páty, Hungary) shown in Figure 10A. All animals were placed between the circuit’s plane-parallel asymmetric electric condensers during treatment. The circuit consisted of a 72-cm^2^ aluminum electrode which served also as a heating pad used to maintain the physiological temperature of the animal at approximately 37 °C (Figure 10B). The upper DIA16 conductive textile chest electrode was placed on the chest, overlying the tumor-burdened lungs. In pilot experiments, the conventional round upper electrode used mainly in the treatment of primary solid tumors in mice (Figure 10C) was tested to verify whether it can induce lung-specific hyperthermia with the potential for application in the treatment of lung tumors in mice. However, this was observed to induce non-lung-specific hyperthermia, with significant dissipation of energy to adjacent non-target tissues and organs in mice thorax, thus making it inadequate for the purpose of this study. Treatment with the lung-optimized chest electrode (Figure 1B) was repeated every third day for a total of six times. Animals were anesthetized with 2% isoflurane during treatment. Animals were housed separately, and treated and control animals were maintained under similar conditions. Animals tested in this study were the offspring of C57BL/6 colonies grown in the animal facility of Semmelweis University, Budapest, Hungary. All animal work conducted during this study was approved by the Governmental Ethical Committee under the permission number PE/EA/51-2/2019.

### 4.4. In Vitro mEHT Treatment

B16F10 melanoma cells were grown on coverslips coated with poly-L-lysine (Sigma-Aldrich) and treated accordingly for 30, 90 min at a temperature range of 41.5–42.0 °C between two plane-parallel electric condenser plates using LabEHY-200 device (Oncotherm Kft, Budaörs, Hungary). Experiments were repeated at least three times. Until further processing, coverslip cell cultures were placed in a fresh culture medium.

### 4.5. Immunohistochemistry

Lung tissues fixed in 10% neutral buffered formalin were dehydrated and embedded in paraffin. Approximately 2.5 µm thick serial sections were cut, mounted on silanized glass slides, and kept in a thermostat at 65 °C for 1 h. Sections were dewaxed and rehydrated for hematoxylin–eosin (H&E) staining, Masson’s trichrome staining, and immunohistochemistry (IHC). For antigen retrieval, sections were heated for 20 min in Tris-EDTA (TE) buffer pH 9.0 (0.1 M Tris_base and 0.01 M EDTA) using an Avair electric pressure cooker (ELLA 6 LUX (D6K2A), Bitalon Kft, Pécs, Hungary) followed by a 20 min cooling with an open lid. Endogenous peroxidases were blocked using 3% H_2_O_2_ in methanol while non-specific proteins were blocked with 3% bovine serum albumin (BSA, #82-100-6, Millipore, Kankakee, IL, USA) diluted in 0.1 M Tris-buffered saline (TBS, pH7.4) containing 0.01% sodium-azide, both for 15 min. The sections were incubated with primary antibodies (Table 1) diluted in 1% BSA/TBS+TWEEN (TBST, pH 7.4) overnight in a humidified chamber. Peroxidase conjugated anti-rabbit and anti-mouse IgGs (HISTOLS-MR-T, micropolymer-30011.500T, Histopathology Ltd., Pécs, Hungary) were used for 40 min incubations and the enzyme activity was revealed by 3,3′-diaminobenzidine (DAB) chromogen/hydrogen peroxide kit (DAB Quanto, #TA-060-QHDX, Thermo, WA, USA) under microscopic control. All incubations were done at room temperature, with samples washed between incubations in TBST buffer for 2 × 5 min. Digitalization of slides and evaluation of immune reactions were done using modules of the QuantCenter image analysis software tool pack (3DHISTECH, Budapest, Hungary). As multiple pulmonary melanoma nodules were noted in each lung sample, all tumors in each section were annotated for subsequent evaluations. The portion of *p*-H2Axγ (Ser139), CCasp3, and p21^waf1^, Ki67 (SP6), F4/80 (D2S9R) XP, CD3, CD8α (D4W2Z) XP-positive cells were quantified as a percentage of the total annotated tumor areas (HistoQuant module), while the portion of MPO-positive cells and Masson’s trichrome-positive area was quantified as a percentage of the total lung area. Evaluation of histological lung injury was performed as described by Ehrentraut et al. [38] taking into consideration scores of individual components of cellular inflammatory cell infiltrates in air space or vessel wall (1 = only wall; 2 = one to five cells (few cells) in air space; 3 = intermediate; 4 = severe (congestion of air space)), interstitial congestion and hyaline membrane formation (1 = normal lung; 2 = moderate (<25% of lung section); 3 = intermediate (25–50% of lung section); 4 = severe (>50% of lung section)), hemorrhage (0 = absent; 1 = present). From each animal, a total of six representative images were analyzed using a blinded semi-quantitative scoring system.

### 4.6. Flow Cytometry Analysis of Apoptotic and Necrotic Cell Death

For detection of apoptotic and/or necrosis in treated tumor cells using flow cytometry, cells were stained with FITC Annexin V Apoptosis Detection Kit with 7-AAD (BioLegend, San Diego, CA, USA). Stained cells were analyzed using a fluorescence-activated cell sorting (FACS) Calibur (BioLegend, San Diego, CA, USA).

### 4.7. Statistical Analysis

Statistical analysis was performed using GraphPad Prism software (v.6.07; GraphPad Software Inc., La Jolla, CA, USA). The normality of data distribution was assessed using the Kolmogorov–Smirnov test and unpaired *t*-test or Mann–Whitney nonparametric test was performed accordingly for determination of statistical significance. Data are expressed as mean ± SEM, *p* < 0.05 were considered significant.

## 5. Conclusions

In this study, we demonstrated that mEHT treatment (at 41.5–42.0 °C for 30 min) inhibits tumor growth and spontaneous proliferation of B16F10 melanoma in a mouse pulmonary metastasis model. A significant decrease in metastatic pulmonary melanoma nodules and lung weight were observed in treated animals. Although a marked decrease in tumor burden was observed in treated animals, direct mEHT-induced tumor cell death was not evident, suggesting that the beneficial effect of mEHT under the current treatment dose may be due to early spontaneous metastatic growth inhibition, rather than a direct heat-induced tumor necrosis and/or apoptosis. In vitro treatment of B16F10 melanoma at a higher treatment dose demonstrated significantly increased tumor apoptosis and necrosis, suggesting that mEHT-induced cellular death is dose-dependent. The upregulation of phosphorylated H2AX was demonstrated with mEHT treatment, which led via the p53/p21^waf1^ pathway to upregulation and nuclear localization of p21^waf1^, a potent cyclin-dependent kinase inhibitor responsible for tumor senescence and cell cycle arrest. The downregulating effect of mEHT on Ki67, a prominent tumor proliferation marker, reaffirms its ability to block B16F10 melanoma cell proliferation in treated lungs. The massive infiltration of tumors by CD3 and CD8-positive T-lymphocytes and by F4/80 CD11b-positive macrophages indicates the ability of mEHT to mobilize the immune response in treated animals. However, whether the tumor-infiltrating lymphocytes are actively involved in tumor cell destruction or are in a suppressed state is yet to be investigated. mEHT treatment of non-tumor-bearing mice demonstrated no evidence of chronic inflammation, fibrotic lesions, or lung injury, emphasizing the safety and lack of auxiliary lung damage that may be associated directly with mEHT treatment. Despite the obvious therapeutic effects of mEHT treatment on the pulmonary metastases of melanoma as described in this paper, it remains to be evaluated whether the synergistic advantages of combining mEHT with already existing treatment modalities may offer a better treatment outcome in the treatment of primary or metastatic pulmonary cancers.

## Figures and Tables

**Table 1:** Antibodies and conditions used for immunohistochemistry.

Antigen	Type	Reference No.	Dilution	Antigen Retrieval	Vendor
p-H2Axγ (Ser139)	Rabbit, mAb	#9718	1:200	T-E	Cell Signaling
CCasp3	Rabbit, pAb	#9664	1:100	Citrate	Cell Signaling
P21^waf1^	Rabbit, mAb	#ab188224	1:300	T-E	Abcam
Ki67 (SP6)	Rabbit, mAb	#MA5-14520	1:100	T-E	Thermo
F4/80 (D2S9R) XP	Rabbit, mAb	# 70076	1:300	T-E	Cell Signaling
CD3	Rabbit, pAb	#IS503	1:3	T-E	Dako
CD8α (D4W2Z) XP	Rabbit, mAb	#98941	1:500	T-E	Cell Signaling
MPO	Goat, pAb	#AF3667	1:300	T-E	R&D Systems

Vendor specification: Cell Signaling (Danvers, MA, USA); Thermo (Waltham, MA, USA); Dako (Glostrup, Denmark); Abcam (Cambridge, UK); Sino Biological (Beijing, China); R&D Systems (Minneapolis, MN, USA); T-E: Tris-EDTA, pH 9.0.
